# ASCO 2018: highlights of urothelial cancer and prostate cancer

**DOI:** 10.1007/s12254-018-0422-0

**Published:** 2018-07-24

**Authors:** Renate Pichler, Wolfgang Horninger, Isabel Heidegger

**Affiliations:** Department of Urology, Medical University Innsbruck, Anichstreet 35, 6020 Innsbruck, Austria

**Keywords:** Prostate cancer, Bladder cancer, Urothelial carcinoma, PARP inhibitors, Immunotherapy, Neoadjuvant chemotherapy

## Abstract

Prostate cancer and urothelial carcinoma are the two most common urological cancers. The aim of this short review is to highlight abstracts from this year’s ASCO Annual Meeting. The phase III SPCG-13 trial showed no difference in biochemical disease-free survival by the addition of docetaxel after primary radiation therapy of localized high-risk prostate cancer. In bone dominant metastatic castration resistant prostate cancer, the phase II radium-223 dose escalation study concluded that the currently used dose with 6 cycles of 55 kBq/kg remains the standard of care. The PARP inhibitor olaparib plus abiraterone provided a significant benefit in radiological progression-free survival compared with abiraterone alone, independent of homologous recombination repair (HRR) mutation status. In localized muscle-invasive urothelial carcinoma, two phase II trials (ABACUS and PURE-01) exploring the pathological complete remission rate of atezolizumab and pembrolizumab prior to cystectomy in cisplatin-unfit or cisplatin-fit patients are presented. Novel targeted therapies such as fibroblast growth factor receptor (FGFR) inhibitors or monoclonal antibodies against nectin-4 confirmed astonishing objective response rates in heavily pretreated metastatic urothelial carcinoma (mUC) patients, resulting in a median overall survival (OS) up to 13.8 months. Finally, updated 1‑year and 2‑year OS survival rates of pembrolizumab and atezolizumab in the first line setting of mUC are presented.

*Prostate cancer* and *urothelial cancer *are the two leading urological tumor entities [1, 2]. Consequently, multiple clinical studies are ongoing either to cure patients with localized disease or to delay tumor progression in advanced stages of the disease.

Even at the latest ASCO 2018 meeting a large number of clinical studies were reported, with clinical practice changing studies in the near future especially in bladder cancer.

## Localized prostate cancer

### Adjuvant docetaxel after primary radiation therapy

The authors of the SPCG-13 trial presented data from a phase III randomized study analyzing the impact of adjuvant docetaxel therapy after primary radiation (≥74 Gy) in patients with localized prostate cancer. All patients were required to harbor an intermediate (PSA 10–20 ng/ml or biopsy Gleason score 7 or cT2b–cT2c) or high risk (PSA > 20 ng/ml or biopsy Gleason score 8–10 or ≥cT3a) stage of the disease. In addition to the routinely used androgen deprivation therapy, all patients were randomized either to 6 cycles docetaxel (75 mg/m^2^) or to placebo after radiation. The primary endpoint of the study was biochemical recurrence defined as a rising PSA ≥ 2 ng/ml above the nadir PSA value.

Although promising former studies on this issue, statistical analyses including 378 patients revealed no significant difference in biochemical disease-free survival in both arms at 5‑year follow-up (progression: +docetaxel vs. surveillance: 31% vs. 30.3%, *p* = 0.631), [3]. To summarize, the present study showed that adjuvant docetaxel treatment does not improve biochemical disease-free survival after radiotherapy in intermediate- or high-risk prostate cancer. However, final results of the RTOG0521 study [4] as well as the subanalysis of the STAMPEDE trial [5] also investigating this topic have to be awaited before drawing any final conclusion of the impact of adjuvant docetaxel therapy in patients with intermediate- or high-risk prostate cancer.

## Metastatic castration resistant prostate cancer (mCRPC)

**Fig. 1:**
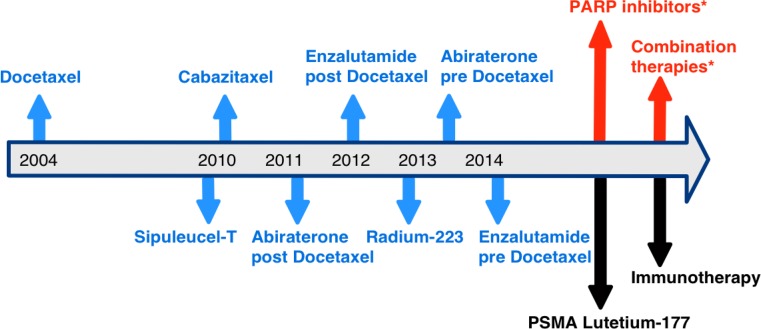
Overview of approved agents in metastatic castration resistant prostate cancer (*blue arrows*) including their year of approval. Future new treatment options are highlighted in *black*, treatment options discussed in this manuscript are marked in *red* with *. *PARP* poly ADP ribose polymerase, *PSMA* prostate-specific membrane antigen

Androgen deprivation therapy is an important backbone treatment in advanced/metastatic prostate cancer; however, most patients will develop a castration-resistant status after 2–3 years. According to the current EAU guidelines castration resistant prostate cancer (CRPC) is defined as serum testosterone < 50 ng/dL or 1.7 nmol/L plus (1) biochemical progression (3 consecutive PSA rises one week apart resulting in two 50% increases over the nadir, and a PSA > 2 ng/mL) or (2) radiological progression (new lesions either 2 or more new bone lesions on bone scan or a soft tissue lesion) [6]. In recent years, several new treatment options have been approved for this stage of the disease (Fig. 1); however, recent data from a hospital-based registry revealed that these new agents since 2010 showed a modest benefit on overall survival rates in metastatic CRPC patients, with a median improvement of 6 months [7].

Therefore, there is need of improvement of existing therapies, development of new therapeutic agents as well as gain of a better knowledge about combining approved and upcoming therapeutic agents.

### Radium-223 dosis escalation

According to the pivotal study published several years ago radium-223 is administered for 6 cycles with a dose of 55 kBq/kg in patients with bone dominant metastatic CRPC [8].

At the recent ASCO meeting, Sternberg et al. presented data of a phase II study comparing the standard radium-223 dose versus a high dose (88 kBq/kg for 6 cycles) as well as versus an increase of treatment cycles from 6 to 12 (55 kBq/kg for 12 cycles). Primary endpoint of the study was the symptomatic skeletal event-free survival. Data clearly showed after enrollment of 381 patients no difference in symptomatic skeletal event-free survival among the treatment groups. However, in both treatment arms with extended radium-223 treatment higher incidences of grade 3 treatment-related adverse events were observed [9].

Therefore, the authors concluded that the currently used doses of 55 kBq/kg up to 6 cycles remain standard of care in patients with symptomatic bone dominant metastatic CRPC without any >3 cm lymph node or visceral metastases [9].

### Radium-223 plus enzalutamide

Both radium-223 [10] and the androgen receptor inhibitor enzalutamide [11, 12] are therapeutic options in patients with bone dominant metastatic CRPC.

Maughan et al. presented safety data from a phase II randomized trial of 49 patients treated with the combination of radium-223 plus enzalutamide versus enzalutamide alone. Interestingly they observed no difference in serious adverse events regardless of attribution between two arms [13].

This finding is in contrast to a phase III study (ERA 223, NCT02043678) combining radium-223 plus the CYP17 inhibitor abiraterone. Several months ago, the EMA stopped this trial because 34.7% of patients treated with radium-223/abiraterone had died so far, compared with 28.2% of patients given abiraterone monotherapy. Fractures were also occurred more frequently with the radium-223 combination than the placebo combination (26% vs. 8.1%). Until now the reasons for the increased number of deaths in the combination arm remains speculative.

### PARP inhibition

In 2017 Mateo et al. reported in a phase II study that the PARP inhibitor olaparib significantly increased overall survival in patients no longer responding to standard treatments who had defects in DNA repair genes [14]. To further increase the efficacy a phase II study combining olaparib with abiraterone has been conducted whose mechanistic rationale is a previous preclinical study that PARP is involved in androgen receptor transcription [15].

At the ASCO meeting Clarke et al. reported data of 140 patients randomized either to olaparib monotherapy or to the combination olaparib/abiraterone. The primary endpoint of the study was the radiologic progression-free survival (rPFS). Fortunately, the combined treatment prolonged rPFS from 8.2 to 13.2 months (*p* = 0.034) with a HR of 0.65. Interestingly a subgroup analysis showed that patients without homologous recombination repair mutations also benefited from therapy. Despite highly promising response data, one must be concerned that in the combination arm significantly higher numbers of grade 3 treatment-related adverse events mainly focusing on cardiac events were observed [16].

## Localized muscle-invasive bladder cancer (MIBC)

### Checkpoint inhibitors in the neoadjuvant setting prior to radical cystectomy

Cisplatin-based neoadjuvant chemotherapy followed by radical cystectomy is currently the gold standard in localized MIBC according to the EAU guidelines [17]. Cisplatin-based neoadjuvant chemotherapy achieves pathological complete response (pCR) rates in about 30% [18], resulting in a median 5‑year overall survival (OS) benefit of 5–8% and a 16% reduction in mortality risk [19, 20]. Nevertheless, chemotherapy-associated toxicities, delayed cystectomy, no available biomarkers and the fact that more than 50% of patients are not eligible for cisplatin are reasons for low referral and treatment rates of neoadjuvant chemotherapy [21], although neoadjuvant chemotherapy is not associated with higher perioperative morbidity or mortality [22]. At the ASCO meeting, preliminary results of two phase II trials using atezolizumab [23] (ABACUS) and pembrolizumab [24] (PURE-01) in the neoadjuvant setting were presented. A comparison of these two trials is shown in Table 1. In summary, overall pCR rates were comparable to neoadjuvant chemotherapy, which enriched to 50% in PD-L1 positive patients, and to 90% in PD-L1 positive patients with additional DNA damage repair (DDR) or retinoblastoma (RB1) genomic alteration. Sequential biomarker analysis showed a dynamic increase in PD-L1 und CD8 expression with atezolizumab. Using pembrolizumab, T cell-inflamed signatures significantly discriminated pT0 from non-pT0 patients. In summary, neoadjuvant immunotherapy was associated with few side effects, no delayed surgery with similar pCR rates to chemotherapy, being a novel hopeful approach especially in cisplatin-unfit patients.

**Table 1:** Results of the phase II ABACUS and PURE-01 trial testing atezolizumab and pembrolizumab in the neoadjuvant setting prior to radical cystectomy

		ABACUS [23], Abstract #4506	PURE-01 [24], Abstract #4507
Phase		II	II
Study population (*n*)		68	43
Checkpoint inhibitor		Atezolizumab	Pembrolizumab
Number of cycles		2	3
Cisplatin-fit?		Cisplatin-unfit	Cisplatin-fit
Residual tumor after TURB		Yes	Yes
TNM for inclusion		cT2-T4aN0-N1	≤cT3bN0
pCR	Overall	29%	39.5%
	PD-L1+	40% (≥5% IC)	50% (CPS score ≥ 20%)
	PD-L1−	16%	–
			PD-L1+ and DDR/RB1-GA: 90%
			DDR and/or RB1-GA: 60%
Discontinuation/progression during CPI (*n*)		1 (1.5%)	1 (2.3%)
Most common AEs		21% fatigue	11% hyperthyroidism
Biomarkers		PD-L1	CPS Score, TMB
		CD8	22-gene T‑cell inflamed panel qPCR
			Genomic profiling

*TURB* transurethral resection of the bladder; *pCR* pathological complete response; *CPI* checkpoint inhibitor; *AE* adverse events; *CPS* combined positive score; *DDR* DNA damage repair; *RB1-GA* retinoblastoma genomic alteration; *TMB* tumor mutational burden

## Metastatic urothelial carcinoma (mUC)

### Overall survival (OS) updates of pembrolizumab and atezolizumab in the 1st line setting of cisplatin-unfit patients

Compared to the results of the EORTC 30986 trial by De Santis M et al. [25] that examined two carboplatin-based chemotherapy regimens (gemcitabine/carboplatin and methotrexate/carboplatin/vinblastine) in cisplatin-unfit patients [25], the survival update analysis of the KEYNOTE-052 [26] and IMvigor210 (cohort 1, [27]) studies presented at this ASCO meeting by Balar et al. [28] (Abstract #4523) and Vuky et al. [29] (Abstract #4524) confirmed a better median OS, 1‑year and 2‑year OS rate as shown in Table 2.

**Table 2:** Overview of updated survival data of the KEYNOTE-052 and IMvigor 210 (cohort 1) trial in comparison to results of the EORTC 30986

	KEYNOTE-052 [29], Abstract #4524	IMvigor210 (Cohort 1) [28], Abstract #4523	EORTC 30986 [25], *Gemcitabine/carboplatin*
Study population (*n*)	370	119	238
ORR (%)	28.9	24	36
CR Rate (%)	8.1	8	6.1
Median OS (months)	11.5	16.3	9.3
1-Year OS rate (%)	47.5	58	37
2-Year OS rate (%)	–	41	18

*ORR* objective response rate, *OS* overall survival, *CR* complete response

Nevertheless, according to preliminary data from the ongoing KEYNOTE-361 (NCT02853305) and IMvigor130 trial (NCT02807636) showing reduced survival with pembrolizumab and atezolizumab compared with standard chemotherapy in mUC patients who have not received prior therapy and whose tumors have low PD-L1 expression, the European Medicines Agency (EMA) restricts pembrolizumab and atezolizumab as monotherapy in the first-line setting only for cisplatin-unfit patients with high PD-L1 expression (≥5% for atezolizumab; tumoral CPS score ≥ 10% for pembrolizumab) [30, 31].

### Targeted therapies in chemotherapy- and IO-refractory mUC

Previous trials have shown that responses to chemotherapy and IO vary by The Cancer Genome Atlas (TCGA) molecular subtyping in MIBC [32–34]. The luminal papillary I subtype UC is characterized by FGFR3 alterations [35], confirming no benefit from neoadjuvant chemotherapy prior to cystectomy [34] and being immunologically “cold” with no clear response to checkpoint inhibitors [32, 36]. Thus, patients with luminal I subtype may be ideal candidates for FGFR inhibitors. Results of phase I and II trials evaluating two FGFR inhibitors, rogaratinib [37] (Joerger M et al., Abstract #4513) and erdafitinib [38] (Siefker-Radtke A et al., Abstract #4503), in heavily pretreated mUC patients with FGFR alterations confirmed astonishing objective response rates in up to 40.4%, increasing to 59% in those patients with prior IO. The median OS (13.8 months) for erdafitinib was higher compared to pembrolizumab (KEYNOTE-045: 10.1 months; [39] Fradet T et al., Abstract #4521) and atezolizumab (Imvigor211: 11.1 months [40]; IMvigor210 cohort 2: 7.9 months, [28] Balar et al., Abstract #4523) in the second line setting. Thus, a phase III trial (THOR, NCT03390504) is ongoing randomizing patients for erdafitinib or pembrolizumab or chemotherapy (vinflunine, docetaxel).

Enfortumab, an antibody–drug conjugate that delivers cytostatic drugs to cells expressing nectin-4, a transmembrane cell adhesion molecule which is expressed in 83% of UC [41], showed similar results in a phase I trial (Rosenberg JE et al. [42], Abstract #4504) as erdafitinib concerning ORR and median OS (Table 3). Due to this encouraging preliminary findings, enfortumab will be evaluated in the third line setting compared to chemotherapy (vinflunine, docetaxel, paclitaxel) in a phase III trial in patients who progressed after platinum-based chemotherapy and IO therapy (NCT03474107).

**Table 3:** Results of novel targeted therapies using FGFR inhibitors (rogaratinib, erdafitinib) and nectin-4 monoclonal antibody (enfortumab) in heavily pretreated mUC patients

	ROGARATINIB [37], Abstract #4513 *N* = 51	ERDAFITINIB [38], Abstract #4503 *N* = 99	ENFORTUMAB [42], Abstract #4504 *N* = 112
Phase	I	II	I
Dosage	800 mg twice daily	8 mg daily (up to 9 mg)	1.25 mg/kg (day 1, 8, 15)
Target	FGFR1-4	FGFR1-4	Nectin-4
Inclusion criteria	≧1 line Cx	≧1 line Cx or	≧1 line Cx or
		Cisplatin-unfit	Cisplatin-unfit
	Prior IO allowed	Prior IO allowed	Prior IO allowed
Visceral metastasis	–	79%	77%
Crea Clearance < 60		53%	50%
≧2 lines systemic Cx		43%	63%
Prior IO		23%	79%
FGFR mutations	FGFR3+ 87%	FGFR3 mutation 75%	–
	FGFR1+ 5%	FGFR2/3 fusion 25%	
	Dual FGFR +8%		
ORR			
Overall	24%	40.4%	41%
Prior IO	30%	59%	40%
Visceral metastasis	–	38.5%	39%
Median PFS (months)	–	5.5	5.4
Median OS (months)	–	13.8	13.6
AEs	Diarrhea (60.8%)	Hyperphosphatemia (73%)	Fatigue (54%)
	Hyperphosphatemia (45.1%)	Skin disorders (49%)	Grade ≧ 3 AEs: anemia 8%, hyponatremia 7%, UTI 7%, and hyperglycemia 6%
		Nail disorders (52%)	

*IO* immuno-oncology, *Cx* chemotherapy, *ORR* objective response rate, *FGFR* fibroblast growth factor receptor, *OS* overall survival, *PFS* progression-free survival, *AE* adverse event, *UTI* urinary tract infection
